# Letter to the editor regarding the paper “Tick infestation of the eyelid”

**DOI:** 10.1590/0037-8682-0398-2020

**Published:** 2020-11-13

**Authors:** Filipe Dantas-Torres

**Affiliations:** 1 Fundação Oswaldo Cruz, Instituto Aggeu Magalhães, Departamento de Imunologia, Recife, PE, Brasil.

Dear Editor:

I have read with interest the paper by Varma and collaborators, titled “Tick infestation of the eyelid”, (https://www.scielo.br/scielo.php?script=sci_arttext&pid=S0037-86822020000100821) which has recently been published in *Revista Brasileira de Medicina Tropical* ^1^. The authors reported the finding of a tick on the eyelid of a 57-year-old man. However, the article contains several issues that should be addressed for clarity.

The authors did not mention the country of origin of the patient (India?), which is an extremely relevant information for contextualizing the case report into a broader context. Moreover, they did not explain how the tick was removed or if any treatment was applied, which are important pieces of information in any clinical case report. 

With regard to tick identification, the authors stated that the tick was identified as an engorged adult female “as males do not enlarge upon feeding”. The readers of this prestigious journal should be aware that this is not a valid criterion for differentiating male ticks from female ones. Adult ticks have four pairs of legs and are sexually mature (they have a genital opening). The basic difference between males and females of hard ticks (family Ixodidae) is the dorsal scutum, which is anterior (podonotal scutum) in the females and complete (i.e., it covers the entire dorsal surface of idiosoma; holonotal scutum) in the males. Besides, the larvae (three pairs of legs and no genital opening) and nymphs (four pairs of legs and no genital opening) also engorge upon feeding.

Further, the authors used non-taxonomic criteria to identify the tick species. The tick found is inornate (i.e., no ornamentation on the dorsal scutum) and, unbelievably, they used scutum ornamentation to distinguish the tick found from the American dog tick (*Dermacentor variabilis*) and the lone star tick (*Amblyomma americanum*). In reality, these two ticks occur in North America, not in India (supposed origin of the patient). Therefore, the authors should have used proper morphological criteria and taxonomic keys to identify the tick species, distinguishing it from its congeners present in the patient’s country of origin.

For instance, supposing that the genus of the tick is correct, other *Rhipicephalus* spp. may be found in India, including in humans^2^. Nonetheless, the figures presented are of low quality to make any conclusion in this sense. In the same way, as emphasized previously^3^ ^-^ ^5^, any tick showing morphological features compatible with the species *Rhipicephalus sanguineus* sensu stricto (i.e., in the narrow sense) should be referred to as “*Rhipicephalus sanguineus* sensu lato” in the absence of confirmatory genetic data. Indeed, a previous study suggested the existence of at least two distinct genetic lineages of brown dog ticks in India, which actually are not *R. sanguineus* sensu stricto^6^.

Another problem is that the authors mentioned that the brown dog tick is a vector of “*Rickettsia rickettsia*” [*sic*]. Besides the misspelling of the species epithet (the correct is “*rickettsii*”), this bacterium is present in the western hemisphere (not in India, again, supposed origin of the patient). Furthermore, the authors mention tick paralysis as another “rare manifestation”. Actually, tick paralysis is a common occurrence in some countries (e.g., Australia) and not typically related to brown dog ticks. The article cited by the authors to support their statement did not provide information on tick species or even country of origin of the patient (Turkey?)^7^.

Finally, the instructions to authors for the section Images of Infectious Disease of *Revista Brasileira de Medicina Tropical* advised that figures should be of the best possible quality. Unfortunately, the figures are of low quality and there is a cotton swab in Figure 2, which I suppose was used as a scale bar.

*Revista Brasileira de Medicina Tropical* is a traditional journal in Brazil and contains the history of tropical medicine of this country, along with other journals, such as *Memórias do Instituto Oswaldo Cruz* and *Revista do Instituto de Medicina Tropical de São Paulo*, just to mention a few. In this perspective, I urge authors and reviewers of this journal to increase considerably their level of criticism, ultimately to increase the quality of manuscripts published in this prestigious journal for the benefit of science and to honor the long tradition of the *Revista Brasileira de Medicina Tropical*. 
